# Cre mRNA Is Not Transferred by EVs from Endothelial and Adipose-Derived Stromal/Stem Cells during Vascular Network Formation

**DOI:** 10.3390/ijms22084050

**Published:** 2021-04-14

**Authors:** Jaana Schneider, Marianne Pultar, Johannes Oesterreicher, Madhusudhan Reddy Bobbili, Severin Mühleder, Eleni Priglinger, Heinz Redl, Andreas Spittler, Johannes Grillari, Wolfgang Holnthoner

**Affiliations:** 1 AUVA Research Centre, Ludwig Boltzmann Institute for Experimental and Clinical Traumatology, 1200 Vienna, Austria; jaana.schneider@trauma.lbg.ac.at (J.S.); marianne.pultar@trauma.lbg.ac.at (M.P.); johannes.oesterreicher@trauma.lbg.ac.at (J.O.); madhusudhan.bobbili@trauma.lbg.ac.at (M.R.B.); eleni.priglinger@trauma.lbg.ac.at (E.P.); heinz.redl@trauma.lbg.ac.at (H.R.); johannes.grillari@trauma.lbg.ac.at (J.G.); 2 Austrian Cluster for Tissue Regeneration, 1200 Vienna, Austria; andreas.spittler@meduniwien.ac.at; 3 Centro Nacional de Investigaciones Cardiovasculares (CNIC), Molecular Genetics of Angiogenesis Group, 28029 Madrid, Spain; severin.muhleder@externo.cnic.es; 4 Department of Surgery, Research Labs & Core Facility Flow Cytometry, Medical University of Vienna, 1090 Vienna, Austria; 5 Department of Biotechnology, Intitute of Molecular Biotechnology, BOKU-University of Natural Resources and Life Sciences, 1180 Vienna, Austria

**Keywords:** extracellular vesicles, endothelial cells, cell–cell communication, Cre-loxP system, coculture

## Abstract

Coculture systems employing adipose tissue-derived mesenchymal stromal/stem cells (ASC) and endothelial cells (EC) represent a widely used technique to model vascularization. Within this system, cell–cell communication is crucial for the achievement of functional vascular network formation. Extracellular vesicles (EVs) have recently emerged as key players in cell communication by transferring bioactive molecules between cells. In this study we aimed to address the role of EVs in ASC/EC cocultures by discriminating between cells, which have received functional EV cargo from cells that have not. Therefore, we employed the Cre-loxP system, which is based on donor cells expressing the Cre recombinase, whose mRNA was previously shown to be packaged into EVs and reporter cells containing a construct of floxed dsRed upstream of the eGFP coding sequence. The evaluation of Cre induced color switch in the reporter system via EVs indicated that there is no EV-mediated RNA transmission either between EC themselves or EC and ASC. However, since Cre mRNA was not found present in EVs, it remains unclear if Cre mRNA is generally not packaged into EVs or if EVs are not taken up by the utilized cell types. Our data indicate that this technique may not be applicable to evaluate EV-mediated cell-to-cell communication in an in vitro setting using EC and ASC. Further investigations will require a functional system showing efficient and specific loading of Cre mRNA or protein into EVs.

## 1. Introduction

Vascularization of engineered tissues remains one of the most important issues to be addressed in tissue engineering and regenerative medicine. Vascular network formation in vitro is predicated on the observation that endothelial cells (EC) form tubular networks when cultivated under appropriate conditions [1,2,3,4,5]. As we previously reported, EC need the proximity of stabilizing mesenchymal stromal/stem cells (MSCs) to form vascular networks [2,3,6]. Additionally, adipose tissue-derived MSC (ASC) release proangiogenic and regulatory proteins [6]. However, it is important to elucidate the molecular pathways and cellular interactions during functional vessel formation. It is known that the physiologically collocated pericytes (PCs) and EC utilize direct cell-to-cell contact and paracrine signaling for intercellular communication [7,8]. In the past decade, extracellular vesicles (EVs) emerged as novel mediators of such intercellular communication by delivering their cargo to recipient cells in a paracrine and autocrine manner [9,10]. Through random but also specific cargo loading, release, and subsequent binding of content, EVs are capable to affect the phenotype and (patho) physiological condition of recipient cells [9,11]. These complex processes of EV–cell interaction and cargo transfer are still not fully elucidated and strongly rely on the biogenesis and origin of the EVs and on the type of recipient cell [9,10,12].

In general, small EV biogenesis is described as formation of intraluminal vesicles during multivesicular body (MVB) maturation within the endosomal system, which involves sorting machineries such as the endosomal sorting complex required for transport (ESCRT) [12,13]. On the other hand, microvesicles are formed via outward budding and scission directly from the plasma membrane [9,13,14]. Since classification by biogenesis is very difficult, EV subtypes are rather defined through their physical characteristics, e.g., their density and size (“small EVs” and “intermediate/large EVs”) [15]. The different subtypes of EVs vary also in their transported cargo. One of the most abundantly packaged molecules is different types of RNAs. Several studies indicated that particularly RNA binding proteins (RBP) are involved in the selective loading of RNA into EVs [11,16]. Following loading and secretion, EVs first bind to the recipient cell surface, which can be mediated by different molecule interactions involving, e.g., integrins and tetraspanins [10]. EVs can either stay bound at the plasma membrane, or they are internalized via different ways of endocytosis [9,17]. After the uptake, EVs follow endosomal pathways leading to internalization into MVB and ending mainly in lysosomal degradation [18,19]. However, it was shown that via back fusion with the MVB membrane the EV-cargo could be released into the cytoplasm as well [9,20].

Since MSC are widely used in numerous tissue engineering approaches [4,21,22], several studies investigated EVs secreted by MSC [23,24,25]. MSC are already known for their proangiogenic and therapeutic potential. Recent evidence shows that small MSC-derived EVs promote EC in the formation of tubular structures, thus promoting angiogenesis [23,24].

It is known that EVs released from different cell types play an important role in vascular network formation and their bioactivity is strongly affected by the cargo and the status of the cell of origin. However, this complex intercellular communication system needs to be further investigated by utilizing different methods to measure cellular uptake of functional cargo transfer via EVs. Therefore, the aim of this study was to investigate if the proangiogenic effect of MSC on EC is influenced by intercellular communication via EVs. In order to elucidate this pathway, and the loading and transfer of RNA via EVs, the Cre-loxP system was adapted to discriminate cells, which have received and utilized functional cargo via EVs from cells that have not. We investigated EV-mediated cargo transfer using flow cytometry and fluorescence microscopy. We could not detect Cre mRNA in the EVs of human umbilical vein endothelial cell (HUVEC) Cre^+^, human induced pluripotent stem cell derived endothelial colony forming cells (hiPSC-ECFCs) Cre^+^, and ASC Cre^+^, thus functional cargo transfer was not observable. Additionally, stimulation of HUVEC with tumor necrosis factor-α (TNF-α) did not result in any detectable change in Cre mRNA loading into EVs and increased vesicle release. However, TNF-α stimulation showed a difference in the abundance of CD81 harboring EVs derived from HUVEC.

## 2. Results

### 2.1. Strategy—Adaption of the Cre-loxP System to Evaluate EV Uptake

**Figure 1:**
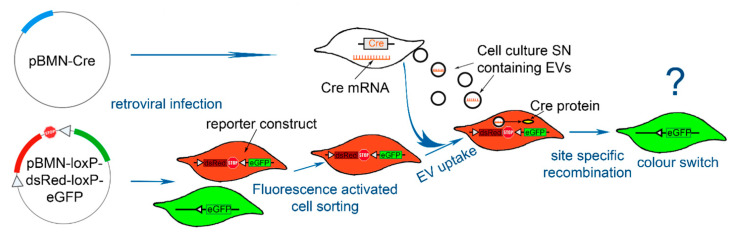
Scheme of the strategy applying the adapted Cre-loxP system for extracellular vesicle (EV) uptake evaluation. The plasmids encoding for the Cre recombinase and the reporter construct were retrovirally transduced into the cells (human umbilical vein endothelial cell (HUVEC), human induced pluripotent stem cell derived endothelial colony forming cell (hiPSC-ECFC), and adipose tissue-derived mesenchymal stromal/stem cell (ASC)). Reporter cells expressing the complete reporter construct (red) consisting of a floxed dsRed cDNA and a stop codon upstream of an eGFP cDNA were sorted by fluorescence activated cell sorting (FACS) for dsRed expression. Cre expressing cells (white, Cre^+^) are producing EVs containing Cre mRNA, which is introduced into the reporter cells via EV uptake. Due to site specific recombination the floxed part of the reporter construct is deleted and a color switch to eGFP (green) is detectable.

In order to distinguish between reporter cells, which have taken up functional EV cargo, the Cre-loxP system was adapted as depicted in Figure 1. Reporter cells were retrovirally infected with a construct containing a floxed dsRed cDNA, followed by a stop codon upstream of an eGFP cDNA. Upon uptake of Cre mRNA, delivered by EVs, Cre recombinase is expressed and the floxed dsRed is deleted. Subsequently, the reporter cells switch to eGFP expression as described in detail by Zomer et al. [26].

### 2.2. Functionality Control via Transfection and Superinfection of Reporter Cells with the Cre Construct Shows Cre Mediated Recombination

**Figure 2:**
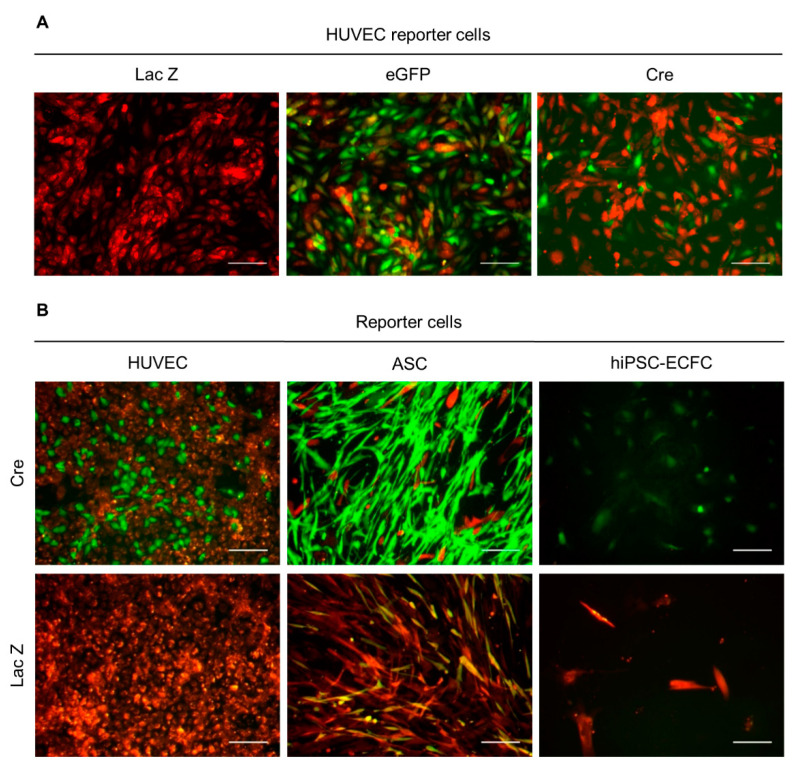
Functionality control of the Cre construct and reporter gene. (**A**) HUVEC reporter cells were retrovirally transduced with a control construct (lacZ), eGFP, and the Cre construct. Data presented were obtained from one experiment. (**B**) HUVEC, ASC, and hiPSC-ECFC reporter cells were transfected with the Cre construct and control construct (lacZ). Data presented was obtained from two independent experiments using two different donors for HUVEC and ASC and one cell donor for hiPSC-ECFC. Scale bar: 100 µm.

In order to assess the functionality of the induced construct, consisting of the floxed dsRed followed by eGFP, reporter cells were either retrovirally infected (superinfection) or transfected with the Cre construct. Approximately 40% of the superinfected reporter cells (HUVEC) showed a change in expression from dsRed to eGFP observable as fluorescent color change from red to green (Figure 2A). The results from the transfection of the reporter cells with pBMN-Cre, as presented in Figure 2B, show that the construct encoding the recombinase was sufficiently effective. eGFP-expressing cells were only detected in Cre transfected cells. Nevertheless, some yellow cells, expressing eGFP and dsRed were visible in control-infected cells of the ASC reporter cells.

### 2.3. Supernatant Change from Cre-Expressing Cells to Reporter Cells—EVs in Conditioned Medium Do Not Mediate Cre Transfer

**Figure 3:**
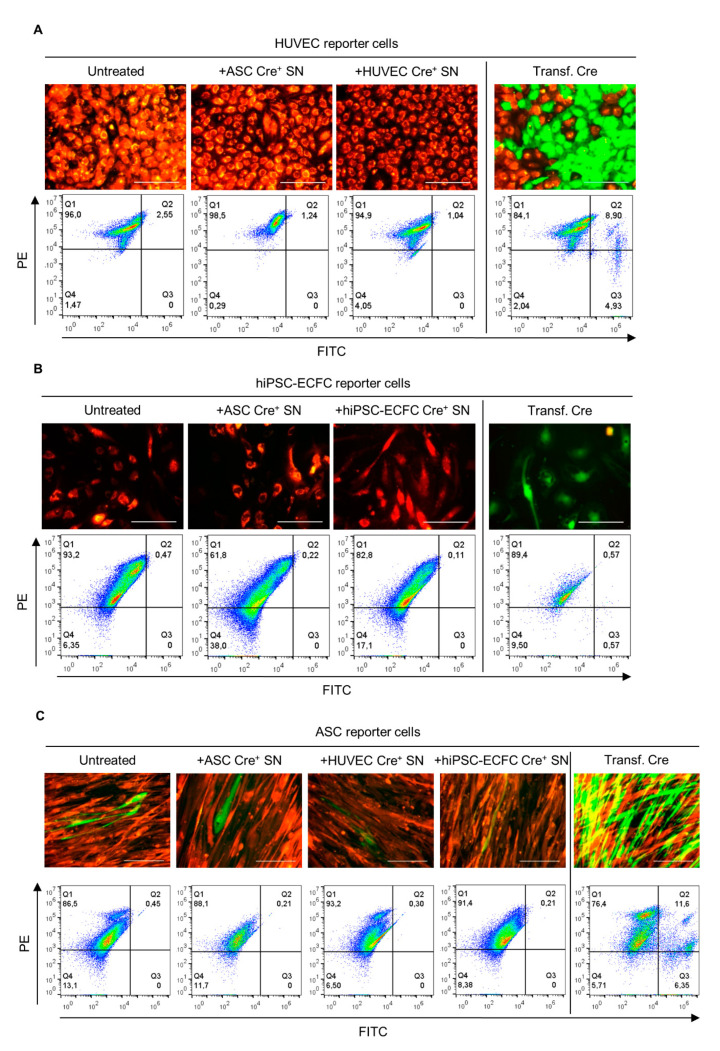
Evaluation of supernatant change from Cre^+^ to reporter cells. HUVEC (**A**), iPSC-ECFC (**B**), and ASC (**C**) reporter cells were cultured under different conditions. Reporter cells cultured in full medium were used as negative control (untreated). Furthermore, as positive control reporter cells were transfected with the Cre construct. Supernatant of HUVEC, hiPSC-ECFC, or ASC Cre^+^ cells were used for culturing reporter cells. Quadrant regions show the percentage of cells in each subpopulation either expressing dsRed (PE, Q1), eGFP (FITC, Q3), both (Q2), or non-fluorescent (Q4). Single green cells are indicated by the white arrows. Data presented was obtained from three independent experiments using two different donors for HUVEC and ASC and one cell donor for hiPSC-ECFC. Scale bar: 100 µm.

We then tested the uptake of Cre containing EVs by reporter cells, and if this leads to Cre-mediated site specific recombination, which deletes the floxed part of the reporter construct resulting in a color switch from dsRed (red) to eGFP (green) [26,27]. Therefore, after confirmation of the constructs functionality, the capability of effective cargo transfer including Cre mRNA via EVs was evaluated. Untreated and Cre transfected reporter cells were used as a control. After 21 days, neither with fluorescent microscopy nor flow cytometry a Cre mediated color switch was detectable in the reporter cells (Figure 3A–C) cultured with supernatant from Cre^+^ cells. Single ASC reporter cells expressing eGFP were observed during imaging but not detected by flow cytometry. However, these were also found in the untreated control.

### 2.4. Direct Coculture of Reporter with Cre^+^ Cells Does Not Induce a Cre-Mediated Recombination

**Figure 4:**
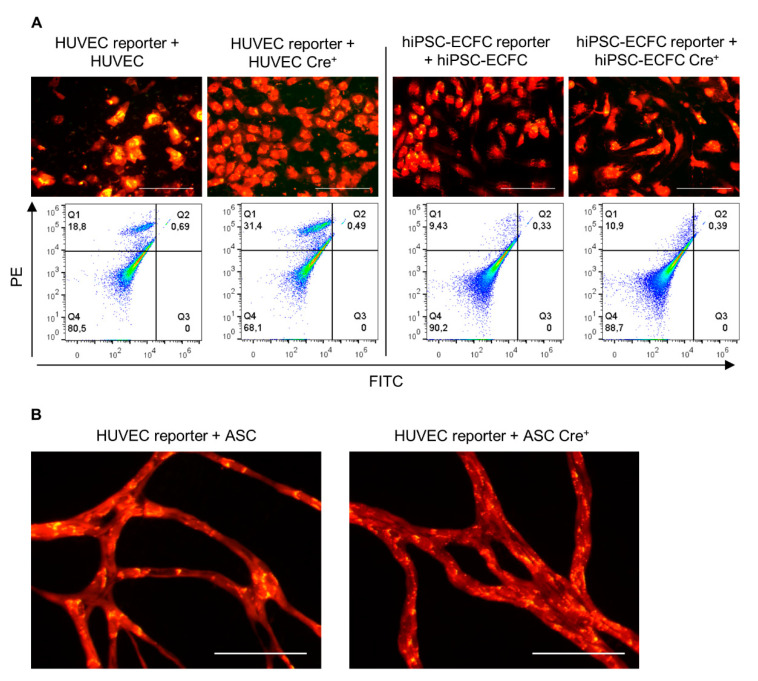
Coculture of Cre^+^ and reporter cells. HUVEC and hiPSC-ECFC reporter cells were either cocultured with Cre^+^ cells of the same cell type or ASC. (**A**) Cocultures of HUVEC and hiPSC-ECFC with Cre^+^ cells or uninfected HUVEC and hiPSC-ECFC (1:10 reporter cells: Cre^+^ cells). Quadrant regions show the percentage of cells in each subpopulation either expressing dsRed (PE, Q1), eGFP (FITC, Q3), both (Q2), or non-fluorescent (Q4). (**B**) HUVEC reporter cells were either cocultured with Cre^+^ or uninfected ASCs (1:50 reporter cells: Cre^+^ cells). Data was obtained from three independent experiments using two different cell donors for HUVEC and ASC and one cell donor for hiPSC-ECFC. *n* = 2 cell donors. Scale bar: 100 µm.

Aiming to investigate the transfer of the recombinase Cre, reporter and Cre^+^ cells were directly cultured together in this experimental setup. As control, non-infected cells of the same type were cocultured with the reporter cells in the same ratio. After 19 days, HUVEC in coculture with ASC show network formation (Figure 4B) but neither with fluorescent microscopy nor flow cytometry a Cre-mediated color switch was detectable in HUVECs or hiPSC-ECFC reporter cells (Figure 4A,B).

### 2.5. Supernatant Transfer from TNF-α Activated and HUVEC Cre^+^ to HUVEC/ASC Reporter Cells Does Not Lead to Cre Mediated Recombination

**Figure 5:**
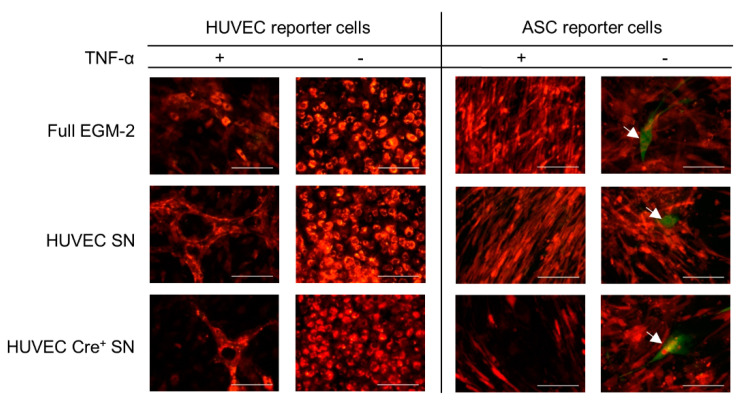
HUVEC and ASC reporter cells cultured under different conditions. Reporter cells were cultured with conditioned medium (supernatant) from HUVEC or HUVEC Cre^+^. Furthermore, the HUVECs used for conditioned medium production were either stimulated with TNF-α (TNF-α +) or unstimulated (TNF-α -). Reporter cells cultured in full medium with or without TNF-α were used as negative control. Single green cells are indicated by the white arrows. Data was obtained from one experiment conducted in technical triplicates. Scale bar: 100 µm.

Since the stimulation of HUVEC with TNF-α has been shown to alter EV release and their content [28,29], functional cargo transport was investigated under these conditions. After 21 days culturing HUVEC or ASC reporter cells with supernatant from TNF-α activated HUVEC Cre^+^ no recombinase mediated color change was observable as shown in the fluorescent micrographs of Figure 5. Single cells expressing eGFP were detected in ASC reporter cells of all culture conditions indicating possible background recombination. Moreover, HUVEC reporter cells cultured with conditioned medium derived from TNF-α activated HUVEC demonstrated formation of network like structures, but not with TNF-α alone. Furthermore, ASC reporter cells were split due to detachment of the cell layer, which was performed six days earlier with ASC reporter cells cultured in the conditioned medium containing TNF-α than that without it.

### 2.6. Detection of Cre in Cells and EVs

**Figure 6:**
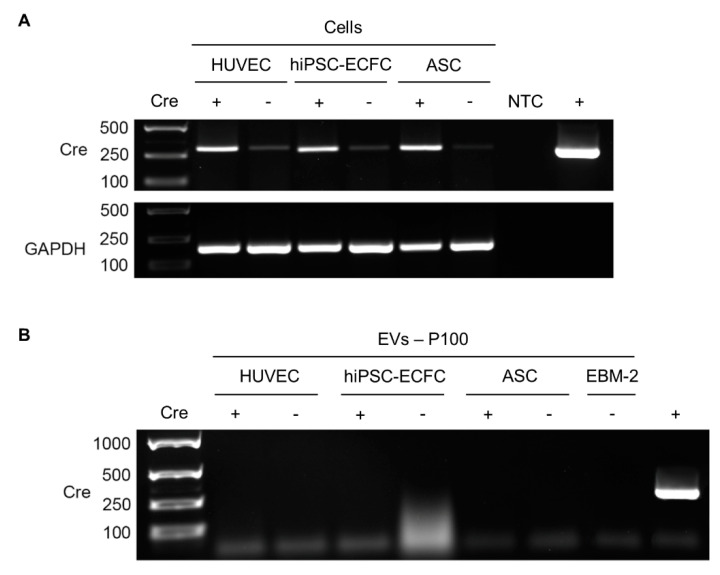
Test for Cre expression in the different cell types and extracellular vesicles (EVs) isolated via ultracentrifugation. (**A**) RNA isolated of the individual cell types retrovirally transduced with pBMN-Cre (Cre^+^) was investigated in comparison to uninfected cells (Cre-) of the same type and donor. GAPDH expression was utilized as control. Furthermore, the plasmid pBMN-Cre was used as positive control (+) and H_2_O as a non-template control (NTC). (**B**) EVs released from Cre^+^ and Cre^−^ cells were obtained via ultracentrifugation (100,000× *g* = P100) and inquired for carrying Cre mRNA. pBMN-Cre was used as positive control (+) and plain EBM-2 as negative control. *n* = 2 cell donors (HUVEC, ASC) and one donor (hiPSC-ECFC).

In order to confirm Cre expression in cells, RNA isolated of the individual cell types retrovirally transduced with the Cre plasmid was examined in comparison to uninfected cells (Figure 6A) of the same type and donor. Glyceraldehyde-3-phosphate dehydrogenase (GAPDH) expression was utilized as a control and was detected in all cellular samples. Furthermore, the plasmid pBMN-Cre was used as positive control (+) and H_2_O as a non-template control (NTC). EVs released from Cre^+^ and Cre^−^ cells were obtained by ultracentrifugation (100,000× *g* = P100) and inquired for carrying Cre mRNA (Figure 6B). Plain endothelial basal medium (EBM-2) was used as a control. We could show that the recombinase is expressed in the retrovirally infected cells but its mRNA was not detectable in their EVs.

### 2.7. Characterization of EVs

**Figure 7:**
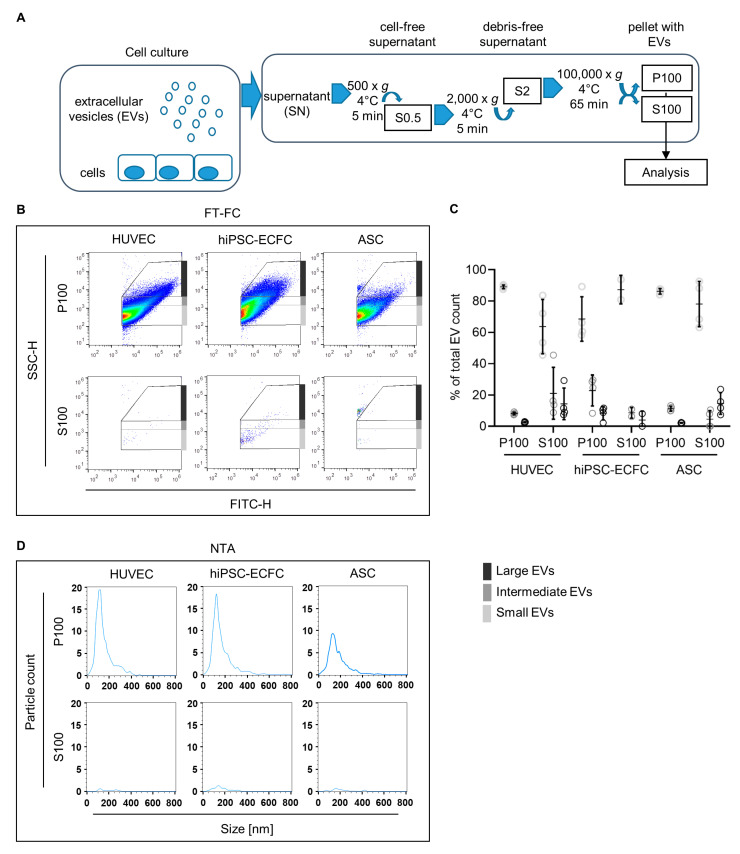
Characterization of particles released from HUVEC, hiPSC-ECFC, and ASC using nanoparticle tracking analysis (NTA) and fluorescence-triggered flow cytometry (FT-FC). (**A**) The obtained cell culture supernatants were cleared from debris and large particle conglomerates via centrifugation at 500 and 2000× *g* for 5 min each. Total EVs, including larger and smaller EVs were enriched by ultracentrifugation for 65 min at 100,000× *g* (P100), which was used to analyze total particle and extracellular vesicle (EV) count via scatter and fluorescence mode nanoparticle tracking analysis (NTA). The total size distribution was assessed via fluorescence-triggered flow cytometry (FT-FC). The remaining supernatant after the differential centrifugation procedure (S100) was analyzed for particle and EV count in order to check the efficiency of the enrichment. (**B**) Resulting representative density scatter plots of FT-FC analyzed EV enrichment show less detected events in the large EV gates (dark grey) for ASC compared to the EC (HUVEC and hiPSC-ECFC). Only a few events could be detected in S100 indicating a successful enrichment. (**C**) Quantification of EV presence in respective size range gates for FT-FC showing increased proportion of “Small” vesicles (light grey) in all cell types and a decrease of “Intermediate” (grey) and “Large” (dark grey) vesicles in P100. Missing percentages to 100% were detected outside the EV gates. Error bars indicate the mean values ± SD (**D**) Size distributions and particle counts of P100 analyzed via scatter mode NTA, for a representative sample of each cell type (HUVEC, hiPSC-ECFC, and ASC). Data was obtained from four independent experiments. *n* = 2 cell donors (HUVEC and ASC) and one cell donor (hiPSC-ECFC).

In order to investigate concentration and size distribution of EVs released by the cells used in this study, particles enriched via ultracentrifugation (Figure 7A) were analyzed using fluorescence-triggered flow cytometry (FT-FC) and nanoparticle tracking analysis (NTA) in the scatter mode. Total P100, enriched for small vesicles, show similar size distribution in all cell types when measured with NTA (Figure 7D). However, the results indicate a lower concentration of released vesicles by ASC compared to HUVEC and hiPSC-ECFC shown by the decreased number of events. Quantification of the FT-FC (Figure 7C) reveals a similar level of smaller (with means of 89% ± 1.2% for HUVEC and 86% ± 1.8% ASC) and larger vesicles (with means of 3% ± 0.4% for HUVEC and 2% ± 0.2% ASC) in HUVEC and ASC. In comparison, hiPSC-ECFC shows an increased presence of larger vesicles (mean of 8% ± 4.3%) and concurrently a decreased presence of smaller vesicles (with a mean of 69% ± 14.2%). Missing percentages to 100% were detected outside the EV gates. As a control of the enrichment procedure S100 was employed, which showed in both, FT-FC and NTA, a significantly diminished amount of events (Figure 7B,C).

### 2.8. Investigation of Concentration and Size of EVs from HUVEC, hiPSC-ECFC, and ASC via NTA and FT-FC

**Figure 8:**
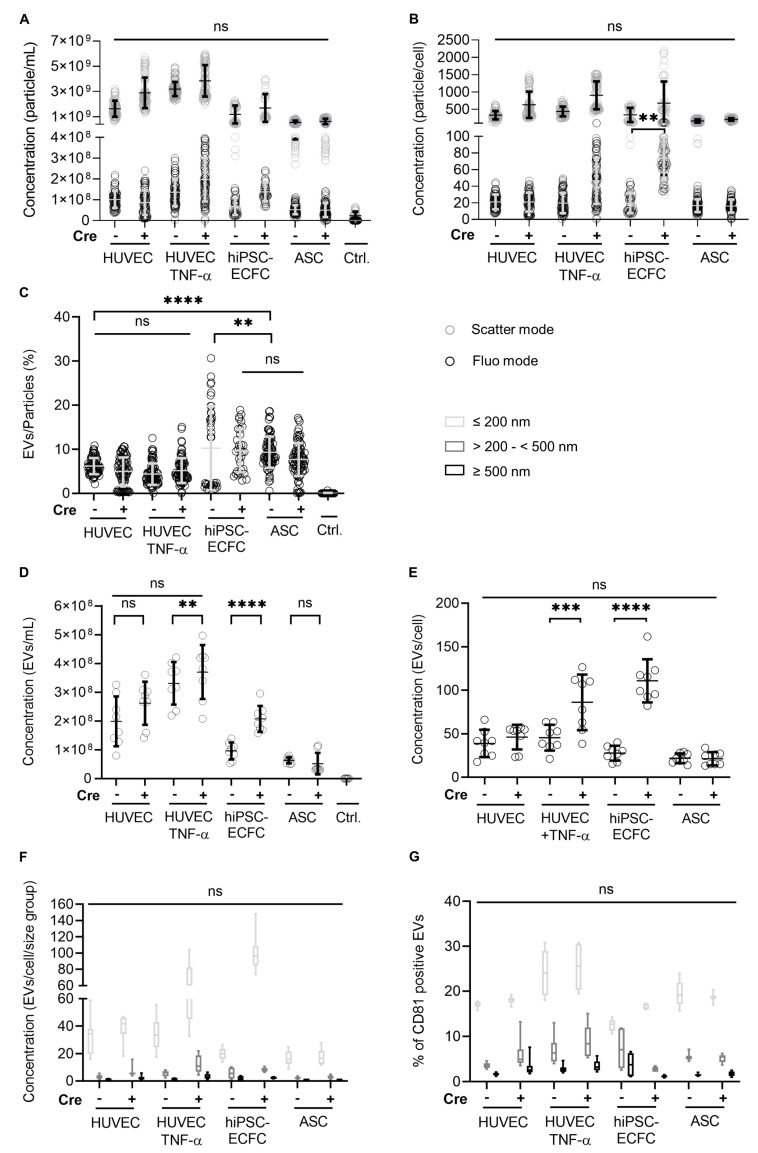
Comparison of extracellular vesicle (EV) release by HUVEC, hiPSC-ECFC, and ASC before and after retroviral transduction with the Cre construct via NTA and FT-FC. (**A**) Concentration (particle/mL) measured in scatter and fluorescence mode NTA showing no significant difference between the different cell types and with or without Cre expression. (**B**) After normalization to cell number a significant difference between hiPSC-ECFC and hiPSC-ECFC Cre^+^ in EVs (fluorescence) release could be detected. (**C**) Calculation of the ratio of EVs (fluorescence mode) and particles (scatter mode) showed a significant difference between endothelial cells and ASC. (**D**) Concentration (particle/mL) measured using FT-FC shows no significant difference between the different cell types and with or without Cre expression. (**E**) After normalization to cell number a significant difference between EC and Cre expressing EC could be detected. (**F**) Size distributions of EVs, analyzed via FT-FC, show no significant difference between the cell types and with or without Cre expression. (**G**) Percentages of CMG-stained and CD81 labeled EVs in the different size ranges (light grey, grey, and dark grey) shows an increase in CD81 positive EVs (<200 nm) released by HUVEC after TNF-α stimulation. **** = *p* < 0.0001, *** = *p* < 0.001, ** = *p* < 0.01, ns = *p* > 0.05. Error bars indicate the mean values ± SD. Data was obtained from four independent experiments. *n* = 2 cell donors (HUVEC, ASC) and one cell donor (hiPSC-ECFC).

In order to estimate the amount of EVs released by the different cells, P100 was measured using NTA and FT-FC. Furthermore, the possible influence of Cre and TNF-α stimulation was investigated with these methods. The analysis of P100 using NTA (Figure 8A–C) showed only a significant difference (*p* < 0.01) between hiPSC-ECFC Cre^−^ and hiPSC-ECFC Cre^+^ in EV (fluorescence) release after normalization to cell number (Figure 8B). As shown in Figure 8C the percentage of EVs (fluorescence) from total particle count (scatter mode) is significantly higher in ASC than HUVEC (with means of 9% ± 3.5% and 6% ± 1.8%, *p* > 0.0001). Measurement of P100 applying FT-FC (Figure 8D–F) showed that after normalization to cell number (Figure 8E) between EC (HUVEC with mean of 39 ± 15.7 EVs/cell and hiPSC-ECFC with mean of 28 ± 8.5 EVs/cell) and ASC (with mean of 22 ± 5.6 EVs/cell) no significant difference was detected. Only hiPSC-ECFC Cre^+^ and HUVEC Cre^+^ + TNF-α showed a significant increase (with means of 111 ± 24.8 EVs/cell for hiPSC-ECFC Cre^+^ and hiPSC-ECFC 28 ± 8.5 EVs/cell, *p* < 0.0001; for HUVEC Cre^+^ +TNF-α 86 ± 31.9 EVs/cell and for HUVEC +TNF-α 46 ± 14.8 EVs/cell, *p* < 0.001) in EV release compared to their non-transfected counterparts. Furthermore, analysis with FT-FC showed similar size distribution (Figure 8F) among the different cell types, except HUVEC Cre^+^ +TNF-α and hiPSC-ECFC Cre^+^ showed an increase in “small” vesicles (≤200 nm) compared to their Cre^−^ counterpart. Additionally, the immunolabeling of the CMG stained particles with the EV-marker CD81 (Figure 8G) showed an increase of CD81 in EVs derived from TNF-α stimulated HUVEC especially in the small vesicle range (≤200 nm) indicating a change in vesicle structure/composition.

## 3. Discussion

The role of EVs in coculture-mediated capillary formation is still poorly understood. Therefore, we adapted the Cre-loxP system to study the EV transfer by discriminating cells taking up EVs from those who do not. The advantages of this technique are that there is no need to isolate the EVs prior to the uptake study, which allowed the direct use of the conditioned medium. Additionally, the functional effect transferred by the EVs should be directly observable, since the reporter cells are clearly distinguishable due to the different fluorescent protein expression (dsRed or eGFP) [26]. Furthermore, EV mediated intercellular communication can be investigated between the specific cell types (ASC, HUVEC, and hiPSC-ECFC). Nevertheless, there are some limitations to this technique. One of them is the inability to detect the amount of EVs taken up by the reporter cells, thus a quantitative evaluation is not possible. Furthermore, it was observed that the packaging of Cre mRNA into EVs varies between different cell types. Hence, each Cre-expressing cell line has to be tested individually. It is also highly recommended to sort the reporter cells and include a negative control consisting of untreated reporter cells since background recombination can never be excluded [26].

In order to evaluate the functionality of the constructs, HUVEC reporter cells were retrovirally superinfected with the Cre containing plasmid. Additionally, the different reporter cells were transfected with the Cre-construct. The functionality control, performing both transfection and superinfection of reporter cells with the plasmid pBMN-Cre, showed a change in protein expression from dsRed to eGFP, thus indicating Cre-mediated and specific site recombination of the reporter construct. However, neither transfer of conditioned medium from Cre^+^ cells to reporter cells, nor coculture of Cre^+^ cells with reporter cells revealed a Cre-mediated recombination thus color change of the reporter cells. Only single ASC showed a color switch, but as these eGFP-expressing cells were also detected in the untreated control, non-specific background recombination was assumed.

Reportedly inflammatory cytokines such as TNF-α increased the release of EVs, which were incorporated by pericytes suggesting an inflammation dependent intercellular communication via EVs [29]. Furthermore, TNF-α has been mentioned to alter EV cargo [28]. Contrary to expectations, upon transfer of supernatant derived from TNF-α activated HUVEC Cre^+^ to reporter cells (ASC and HUVEC), Cre mediated recombination was not detectable. The concentration of EVs released by HUVEC after TNF-α stimulation was also not significantly higher compared to unstimulated HUVEC. Single cells expressing eGFP were observed within ASC of all conditions indicating background recombination (red-to-green color switch without recombinase). Depending on the cell type the frequency of the unspecific recombination varies significantly in reporter cells [26]. This observation corresponds with our results, since we detected background recombination only in ASC but not in hiPSC-ECFC and HUVEC after sorting. However, continuous cultivation of HUVEC with conditioned medium from TNF-α activated HUVEC (supernatant with TNF-α) showed formation of network like structures suggesting that EC activation directly connects angiogenesis and inflammation. As reported by a previous study [30], continuous activation of EC results not only in expression of cellular adhesion molecules and production of reactive oxygen species but also induces formation of angiogenic sprouts without applying angiogenic growth factors. Assuming that continuous stimulation with TNF-α induces sprout formation, the early detachment of ASC, cultured with supernatant from TNF-α activated HUVEC (supernatant with TNF-α), could be associated with the physiological mechanism of PC detaching during angiogenesis.

As mentioned previously, it has to be considered that the packaging of Cre mRNA into EVs presumably differs between cell types [26]. We can assume that different mechanisms lead to RNA release via EVs by the parental cell [11]. These include selective packaging into EVs, mainly facilitated by RBP as suggested by motif enrichment, but also non-selective loading and release directed by the local concentration of RNA [11,16,31]. Therefore, each cell type retrovirally transduced with the Cre construct was evaluated separately for Cre expression. The results from testing cellular Cre mRNA showed weak signals in Cre- cells but a significantly stronger one in the Cre^+^ cells. Furthermore, no Cre mRNA could be detected in the EVs leading to the assumption that Cre is not loaded into the EVs of the used cell types. Nevertheless, EVs derived from Cre^+^ cells contain Cre mRNA and are able to cause EV mediated recombination in reporter cells [27,32]. Since these studies used the adapted Cre-loxP system in vitro only in cancer cell lines [27,32], a comparison to the used primary EC and ASC in this study is difficult. Several reports have shown an increase of EV release and a change in cargo of cancer cells indicating a deregulation of the EV biogenesis. For example, overexpression of ESCRT components or the initiation of oncogenic signaling pathways (e.g., by Epidermal Growth Factor Receptor Variant III) were described to enhance the productions of EVs [33]. Furthermore, oncogenic signaling or hypoxia can lead to altered EV content (protein or RNA) [33,34]. These observed differences in EV biogenesis of cancer cells might influence the mechanistic of the applied Cre-loxP system. However, efficient Cre mRNA packaging and transfer was recently reported between human pulmonary artery smooth muscle cells (HPASMCs) and human pulmonary arterial endothelial cells (HPAECs) in stark contrast to our results [35].

As indicated by the PCR results, we can assume that Cre mRNA is not loaded into EVs of our cell types used here and therefore not transferred to the reporter cells. Thus, further optimization of loading Cre into EVs should be considered for further studies. This could be achieved for example by growing monoclonal Cre^+^ cells from high level expressing clones. Furthermore, the expression vector could be improved by placing the Cre transgene shortly after the promotor and/or introducing zip code sequences guiding Cre mRNA to the EVs [26]. Additionally, we still cannot exclude the possibility that HUVEC do not take up EVs in general.

Evaluation of the particle characteristics (size and concentration) from the different cell types (HUVEC, hiPSC-ECFC, and ASC) by NTA and FT-FC showed successful enrichment of small EVs. Furthermore, the results indicate a release of less “larger” vesicles by ASC in comparison to the EC. On the other hand, a shift to a higher presence of larger vesicles was detectable in hiPSC-ECFC derived EVs. Regarding the concentration of released particles/EVs the different cell types show no significant differences. Only HUVEC Cre^+^ after TNF-α stimulation and hiPSC-ECFC Cre^+^ showed an increase in EV release after normalization to cell number. A reported problem of Cre is that it shows toxic effects [36] leading to deletions or translocations in untargeted genome sites. If this issue could be an explanation for a change in number of released particles/EVs remains to be evaluated.

In summary, this study showed that Cre is not loaded into the EVs of HUVEC, hiPSC-ECFC, and ASC, thus Cre mediated recombination was not detectable, despite possible EV transfer. Stimulation of HUVEC Cre^+^ with the inflammatory cytokine TNF-α again did not show site specific recombination of the reporter construct either. Therefore, further experimental investigations are necessary to confirm the absence of Cre in EVs. Moreover, our results indicate that the currently established Cre-loxP system may not be applicable to investigate transfer of functional biomolecules via EVs during vascular network formation in an in vitro coculture system using primary EC and ASC. Hence, additional improvement of the method might be necessary to ensure the cellular expression and loading of Cre mRNA or protein into EVs.

## 4. Materials and Methods

### 4.1. Cell Culture

The isolation of primary cells was approved by the local ethics committee of the state of Upper Austria with written informed consent by the donors (ethics committee vote #200, 12/05/2005). HUVEC from two different donors were isolated as previously described [3]. hiPSC-ECFC were purchased from Axol Bioscience (Little Chesterford, Cambridge, UK). ASC were isolated as described in [3]. All data shown were obtained from several independent experiments, where two different biological donors were used (indicated by n in each figure) between passage 2 and 10. All cells were cultivated in endothelial growth medium-2 (EGM-2, Lonza, Walkersville, MD, USA). Additional fetal calf serum (FCS, Sigma-Aldrich Co. LLC., St. Luis, MO, USA) was added up to a final concentration of 5%. The final mixture of the medium was designated as “full medium”.

### 4.2. Plasmids and Retroviral Infection of HUVEC, hiPSC-ECFC and ASC

The reporter construct loxP-dsRed-loxP-eGFP and Cre cDNA were subcloned into pBMN vectors using pBMN-lacZ (Addgene, Watertown, Massachusetts, USA), pLV-CMV-loxP-DsRed-loxP-eGFP (a kind gift of Jacco van Rheenen/Johannes Grillari, University of Natural Resources and Life Sciences, Vienna, Austria; Addgene #65726) and pcDNA3.1-CMV-CFP;UBC-Cre25nt plasmid eGFP (a kind gift of Jacco van Rheenen/Johannes Grillari, University of Natural Resources and Life Sciences, Vienna, Austria; Addgene #65727).

The retroviral infection was performed as previously described using Phoenix Ampho cells (kindly provided by Regina Grillari, University of Natural Resources and Life Sciences, Vienna, Austria) [37]. Briefly, virus particles were generated by transfecting Phoenix Ampho cells with Turbofect (Thermo Fisher Scientific Inc., Waltham, MA, USA) according to the manufacturer’s instructions. The supernatants containing virus particles were transferred on HUVEC, hiPSC-ECFC and ASC, and centrifuged onto the cells at 800× *g* for 60 min at RT. Subsequently, all cells were expanded in new cell culture flasks with full medium.

### 4.3. Fluorescence Activated Cell Sorting

Cells retrovirally transduced with the reporter construct were cultured until 100% (HUVEC and hiPSC-ECFC) or 80% (ASC) confluence. After detaching with 1 × TE (Trypsin/EDTA), the cells were strained using a Corning™ Falcon™ test tube with cell strainer snap cap (35 µm, Thermo Fisher Scientific Inc., Waltham, MA, USA). The cells were sorted for dsRed expression by fluorescence activated cell sorting (FACS) with the high-speed 4-way cell sorter FACSAriaTM Fusion (BD Biosciences, Franklin Lakes, NJ, USA).

### 4.4. Imaging

Images were taken with the fluorescence microscope Zeiss Axio Observer A1 Microscope with ICm1 axiocam at the indicated time points of each experiment using 10× and 20× magnification.

### 4.5. Transfection of Reporter Cells

Transfection of cells was performed by seeding 5 × 10^4^ cells/well HUVEC, hiPSC-ECFC, and ASC in a 6-well-plate and culturing them in full medium until reaching approximately 80–90% confluence. Per well 4 µg of each plasmid (pBMN backbone with either Cre, lac Z, eGFP, and floxed dsRed construct) was used for the transfection with Turbofect (Thermo Fisher Scientific Inc., Waltham, MA, USA) performed according to the manufacturer’s instructions. Transfection was examined via fluorescence microscopy after 48 h.

### 4.6. Supernatant Transfer from Cre-Expressing Cells to Reporter Cells

In each experiment two 6-well plates were prepared, one seeded with ASC reporter cells in four of the wells and ASC Cre^+^ in the remaining two wells. The other one was seeded with HUVEC or hiPSC-ECFC reporter and Cre^+^ cells in the same scheme, respectively. ASC were seeded with a density of 3 × 10^4^ cells/well and HUVEC or hiPSC-ECFC with 5 × 10^4^ cells/well. Supernatant of Cre^+^ cells was transferred daily onto reporter cells for 21 days. Reporter cells were analyzed by fluorescent microscopy (images taken at day 2, 6, 10, 15, and 21) and flow cytometry (at day 21).

### 4.7. Direct Coculture of Cre-Expressing Cells with Reporter Cells

HUVEC and hiPSC-ECFC reporter cells were cultured together with their corresponding Cre^+^ cells on a 6-well plate in a ratio of 1:10 (reporter cells: Cre^+^ cells). Furthermore, HUVEC reporter cells were cultured together with ASC Cre^+^ in a ratio of 1:50 (reporter cells: Cre^+^ cells). Cocultures were cultivated for 19 days and analyzed via flow cytometry at day 1, 10, and 19 and fluorescent microscopy (images taken at day 1, 6, 10, 14, and 19).

### 4.8. Supernatant Transfer from TNF-α Activated HUVEC to ASC/HUVEC Reporter Cells

HUVEC and ASC reporter cells were seeded on a 12-well-plate with a density of 2 × 10^4^ cells/well (HUVEC) and 1.2 × 10^4^ cells/well (ASC). HUVEC and HUVEC Cre^+^ were each cultivated in full medium on two T75 flasks until reaching confluency. As a control two T75 flasks with full medium only (w/o cells) were additionally incubated. TNF-α (10 ng/mL) was added to the medium of one of the flasks per cell type and control before medium change and incubated overnight. Supernatant from the HUVEC, HUVEC Cre^+^, and controls was transferred daily to the reporter cells. Images were taken at day 2, 6, 10, 15, and 21. ASC reporter cells were split at day 9 (+TNF-α) and 14 (+TNF-α), when they started to detach.

### 4.9. Analyzing Reporter Cells for Cre Mediated Color Switch via Flow Cytometry

Reporter cells were detached by removing the supernatant, washing the cells with 2 mL 1 × PBS (w/o Ca^++^ Mg^++^) and adding 500 µL Accutase (Sigma-Aldrich Co. LLC., St. Luis, USA) per well. After 5–10 min incubation at 37 °C, the cell suspension was transferred into flow cytometry tubes using 1.5 mL 1 × PBS (w/o Ca^++^ Mg^++^). Cells were centrifuged at 300× *g* for 5 min at RT and the supernatant was discarded. Next, the pellet was resuspended with 1 mL 1 × PBS (w/o Ca^++^ Mg^++^) and the cell suspension was centrifuged at 300× *g* for 5 min at RT followed by discarding of the supernatant. The pellet was resuspended with 250 µL or 500 µL 1 × PBS (w/o Ca^++^ Mg^++^). Finally, the reporter cells were analyzed on their expression of either dsRed or eGFP utilizing the Cytoflex flow cytometer (Beckman Coulter GmbH, Brea, CA, USA). Evaluation of the data was conducted by using the FlowJo V10 software (FlowJo, LLC., Ashland, OR, USA).

### 4.10. Enrichment of EVs via Ultracentrifugation

EVs were collected when cells reached approximately 80% (ASC) or 90% (HUVEC, hiPSC-ECFC) confluency. Full medium was removed and the cells were washed 3× with 1 × PBS (w/o Ca^++^ Mg^++^) to eliminate any contaminating EVs from the FCS. Endothelial Basal Medium (EBM-2, Lonza, Walkersville, MD, USA) was applied and conditioned for 48 h. Two identical flasks of each cell type were cultured using one for EV collection and one to determine cell number prior to conditioning. As a control EBM-2 w/o cells was used. The conditioned medium from each cell type was centrifuged at 500× *g* for 5 min at 4 °C in a fixed angle rotor (SN867, Heraeus Megafuge 16R). The resulting supernatant, termed “S0.5”, was centrifuged again at 2000× *g* for 5 min at 4 °C to remove cell debris. The supernatant (“S2”) was transferred into ultracentrifugation tubes (Ultra-Clear, Beckmann Coulter, CA, USA) and centrifuged at 100,000× *g* for 65 min (including acceleration time) at 4 °C using a swing out rotor (SW40.1 Ti) in the ultracentrifuge L-100XP from Beckman Coulter. The obtained pellet “P100” containing small and large EVs was resuspended in 1 mL sterile-filtered (0.22 µm PVDF filter) 1 × PBS (w/o Ca^++^ Mg^++^). “P100” and an aliquot of supernatant “S100” were stored at 4 °C until further use.

### 4.11. Nanoparticle Tracking Analysis

Size and concentration of EVs were characterized by nanoparticle tracking analysis (NTA). Samples containing EVs obtained via ultracentrifugation (P100) and the supernatant S100 were stained with CellMask Green (CMG, Invitrogen, UK) to distinguish between EVs and non-lipid particles. Therefore, CMG diluted 1:2000 with sterile filtered (0.22 µm PVDF filter) 1 × PBS (w/o Ca^++^ Mg^++^) was added to the samples and incubated for 20 min at 37 °C in an incubator. Subsequently, the samples were further diluted with sterile filtered 1 × PBS (w/o Ca^++^ Mg^++^) to a concentration allowing optimal measurement (according to manufacturer) using the Zetaview PMX110 device from Particle Metrix (Zeta VIEW S/N 239, software ZetaView 8.04.02, camera 0.703 µm/px, Cell S/N: CA0058-0109, Particle Metrix, Meerbusch, Germany) in the fluorescence mode. During sample application the intensity of the laser was lowered to a minimum (shutter 500) to prevent bleaching. Samples were then measured in the fluorescence mode applying shutter 32 and a sensitivity of 95. Samples of the same P100 and S100 were measured using scatter mode with settings of shutter 50 and a sensitivity of 70 to quantify total particle concentration. The data were evaluated with the FlowJo V10 software (FlowJo, LLC., Ashland, OR, USA) and GraphPad Prism 9.0.0 (GraphPad Software, Inc., San Diego, CA, USA).

### 4.12. Analysis of EVs via Fluorescence-Triggered Flow Cytometry

EVs were further analyzed using fluorescence-triggered flow cytometry (FT-FC) as previously described [38]. Briefly, EVs (P100) were stained with CMG and measured via flow cytometry by detecting the particles not based on a scatter signal threshold but on their fluorescence intensity. Thus, only particles with a lipid layer incorporating the green fluorescent dye are detected. Size approximation is based on the side scatter signal of fluorescently labeled silica beads with defined sizes (FITC labeled silica beads 100, 200, and 500 nm, Kisker Biotech, Steinfurt, Germany). Of the resuspended EV pellet (P100) or supernatant (S100) 80 µL were transferred to 1.5 mL tubes, before adding 20 µL of the 1:2000 CMG working solution and incubating for 20 min at 37 °C in the dark. Subsequently, 0.5 µL of the respective directly PE-labeled CD81 antibody (recombinant, Miltenyi Biotec, Clone REA513) and the IgG Isotype (BD Pharmingen, Clone, MOPC-21) diluted in 100 µL filtered (0.22 µm PVDF filter) 1 × PBS (w/o Ca^++^ Mg^++^) were added. After thorough vortexing, the samples were incubated on ice in the dark for 30 min. As controls, 1 × PBS (w/o Ca^++^ Mg^++^) with CMG and/or antibodies and EV samples without CMG and/or antibodies were analyzed (data not shown).

### 4.13. Analysis of Cre Expression

HUVEC, ASC, hiPSC-ECFC, and Cre^+^ cells of the same cell types were grown to confluence on a 6-well plate. After discarding the supernatant, 1 mL per well of Trizol (TRI Reagent; Molecular Research Center, Inc., Cincinnati, OH, USA) was used for lysis. Cell suspension was transferred into a centrifugation tube and 300 µL Chloroform (Panreac AppliChem, Darmstadt, Germany) was added. The mixture was mixed and centrifuged at 4 °C with 12,000× *g* for 15 min. The clear upper phase was taken off and transferred into a new centrifugation tube. RNA was precipitated by adding 500 µL of isopropanol. The tubes were inverted 7 times followed by a further resting time of 10 min at RT. Then the samples were centrifuged at 4 °C with 12,000× *g* for 10 min. The pellet was washed with 70% ethanol (Sigma-Aldrich Co. LLC., St. Luis, MO, USA) and centrifuged at 7500× *g* for 5 min at 4 °C. This washing step was repeated once. Each sample was reconstituted in nuclease-free water (ddH_2_O). RNA concentration was measured by spectrophotometry (Nanodrop OneC, Thermo Fisher Scientific Inc., Waltham, MA, USA).

After removing contaminating DNA from 2 µg RNA via a digestion step using DNAse I (RQ1 RNase-Free DNase, Promega, Madison, WI, USA) according to the manufacturer’s instructions, the RNA samples were precipitated by adding 3 M NaAc-Depc (pH 2.5) and EtOH absolute (−20 °C). The mixture was frozen at −80 °C for 30 min followed by centrifugation at 14,000× *g* for 50 min at 4 °C. The supernatant was discarded and the pellet washed with EtOH (70%, −20 °C). After centrifugation at 14,000× *g* for 5 min at 4 °C the pellet was dried for 10–20 min and reconstituted in ddH_2_O. For the cDNA synthesis the EasyScriptTM cDNA synthesis kit (ABM, Inc. New York, NY, USA) was used according to the manufacturer’s instructions. The synthesis was performed using a thermocycler (T3000, Biometra, Göttingen, Germany). The cDNA was kept at −20 °C. For RNA isolation from EVs one milliliter of Trizol was added to one of the P100 samples from each cell type and proceeded as described above.

PCR was performed to measure the housekeeping gene glyceraldehyde-3-phosphate dehydrogenase (GAPDH) as reference and Cre recombinase. An aliquot of each primer (Table 1) was diluted with ddH_2_O to a final concentration of 10 µM. Subsequently, a PCR master mix using the DNA-Polymerase kit peqGOLD “Hot” Taq-DNA-Polymerase (VWR International, LLC., Solon, OH, USA) was prepared according to the manufacturer’s instructions. Of the template cDNA 100 ng/µL and 5 ng of the plasmid pBMN-Cre as a positive control were used for the PCR using a thermocycler (T3000, Biometra, Göttingen, Germany). In the case of cellular derived cDNA 30 cycles were conducted, whereas for EV-derived samples 40 cycles were conducted. Subsequently, a 1% agarose gel was prepared and the samples, mixed with 6× loading buffer (XCFF; xylene cyanole FF (0.025%), glycerin (30%), and TRIS (10mM)), loaded onto the gel. Of the Quantitas Fast DNA ladder 5 µL (100 bp–2 kb, Biozym Scientific GmbH, Hessisch Oldendorf, Germany) were applied for size evaluation. The gel was run for 20–25 min, at 200 V, 400 mA, and analyzed via ChemiDocTM imaging system (Bio-Rad Laboratories, Inc., Hercules, CA, USA).

### 4.14. Statistical Analysis

The statistical analysis was performed using Graph Pad Prism v.9.0.0 (GraphPad Software, San Diego, CA, USA) and Microsoft Excel 2016 (Microsoft Corporation, Redmond, WA, USA). Outliers were identified by determining if the data points are more than 1.5 interquartile ranges below the first or above the third quartile. Via the D’Agostino–Pearson omnibus test normality was verified. In order to test for significant differences between more than two normally distributed samples, one-way ANOVA was executed, followed by a Tukey’s multiple comparison test. For more than two not-normally distributed groups, the Kruskal–Wallis test and the Tukey’s multiple comparison were performed. The number of used donors (*n*), the *p*-values, and the respective statistical significance are indicated in each figure. The data are plotted as mean with ± standard deviation (SD) and median with the interquartile range.

## Figures and Tables

**Table 1:** Primer sequences.

Primer	Sequence	Source/Manufacturer
Cre sense Cre antisense Cre in sense Cre in antisense Cre sense new Cre antisense new hGAPDH sense hGAPDH antisense	CGACCAGGTTCGTTCACTCA AACACCCTGTTACGTATAGCC CGACCAGGTTCGTTCACTCA AACACCCTGTTACGTATAGCC ATGCTTCTGTCCGTTTGCC CCTGTTTTGCACGTTCACC GAGTCAACGGATTTGGTC GACAAGCTTCCCGTTCTC	Microsynth (Balgach, Switzerland)

## Data Availability

Not applicable.

## Abbreviations

ASC	Adipose tissue-derived MSC
CMG	Cell Mask Green
EBM-2	Endothelial Basal Medium
EGM-2	Endothelial Growth Medium
EVs	Extracellular Vesicles
ESCRT	Endosomal Sorting Complex Required for Transport
FACS	Fluorescence Activated Cell Sorting
FCS	Fetal Calf Serum
FT-FC	Fluorescence Trigger Flow Cytometry
GAPDH	Glyceraldehyde-3-Phosphate Dehydrogenase
hiPSC-ECFC	Human induced Pluripotent Stem Cell derived Endothelial Colony Forming Cells
HUVEC	Human Umbilical Vein Endothelial Cells
MSC	Mesenchymal Stromal/Stem Cells
MV	Microvesicles
MVB	Multivesicular Body
NTA	Nano Particle Tracking Analysis
NTC	Non-Template Control
RBP	RNA Binding Protein
TNF-α	Tumor Necrosis Factor-α
VEGFR-2	Vascular Endothelial Growth Factor Receptor 2
